# Understanding experiences of and preferences for service user and carer involvement in physical health care discussions within mental health care planning

**DOI:** 10.1186/s12888-017-1287-1

**Published:** 2017-04-13

**Authors:** Nicola Small, Helen Brooks, Andrew Grundy, Rebecca Pedley, Chris Gibbons, Karina Lovell, Penny Bee

**Affiliations:** 1 Collaboration for Leadership in Applied Health Research and Care (CLAHRC) for Greater Manchester, Centre for Primary Care, Division of Population Health, Health Services Research and Primary Care, School of Health Sciences, Faculty of Biology, Medicine and Health, Manchester Academic Health Science Centre, University of Manchester, Williamson Building, Oxford Road, Manchester, M13 9PL UK; 2 Division of Nursing, Midwifery and Social Work, Faculty of Biology, Medicine and Health, University of Manchester, Jean McFarlane Building, Oxford Road, Manchester, M13 9PL UK; 3 School of Health Sciences, Faculty of Medicine and Health Sciences, University of Nottingham, Research Office Medical School, Queen’s Medical Centre, Nottingham, NG7 2UH UK; 4 Cambridge Centre for Health Services Research, Institute of Public Health, University of Cambridge, Forvie Site, Robinson Way, Cambridge, CB2 0SR UK; 5 The Psychometrics Centre, Cambridge Judge Business School Executive Education, Cambridge, CB2 1AG UK; 6 Collaboration for Leadership in Applied Health Research and Care (CLAHRC) for Greater Manchester, Division of Nursing, Midwifery and Social Work, School of Health Sciences, Faculty of Biology, Medicine and Health, University of Manchester, Jean McFarlane Building, Oxford Road, Manchester, M13 9PL UK

**Keywords:** Service user involvement, Severe mental illness, Carers, Physical health, Mental health services, Care planning, Qualitative research, Patients’ perspectives, Health outcomes

## Abstract

**Background:**

People with severe mental illness suffer more physical comorbidity than the general population, which can require a tailored approach to physical health care discussions within mental health care planning. Although evidence pertaining to service user and carer involvement in mental health care planning is accumulating, current understanding of how physical health is prioritised within this framework is limited. Understanding stakeholder experiences of physical health discussions within mental health care planning, and the key domains that underpin this phenomena is essential to improve quality of care. Our study aimed to explore service user, carer and professional experiences of and preferences for service user and carer involvement in physical health discussions within mental health care planning, and develop a conceptual framework of effective user-led involvement in this aspect of service provision.

**Methods:**

Six focus groups and four telephone interviews were carried out with twelve service users, nine carers, three service users with a dual service user and carer role, and ten mental health professionals recruited from one mental health Trust in the United Kingdom. Data was analysed utilising a thematic approach, analysed separately for each stakeholder group, and combined to aid comparisons.

**Results:**

No service users or carers recalled being explicitly involved in physical health discussions within mental health care planning. Six prerequisites for effective service user and carer involvement in physical care planning were identified. Three themes confirmed general mental health care planning requirements: tailoring a collaborative working relationship, maintaining a trusting relationship with a professional, and having access to and being able to edit a living document. Three themes were novel to feeling involved in physical health care planning discussions: valuing physical health equally with mental health; experiencing coordination of care between physical-mental health professionals, and having a physical health discussion that is personalised.

**Conclusions:**

High quality physical health care discussions within the care planning process demands action at multiple levels. A conceptual framework is presented which provides an evidence-based foundation for service level improvement. Further work is necessary to develop a new patient reported outcome measure to enable meaningful quantification of health care quality and patient experience.

**Electronic supplementary material:**

The online version of this article (doi:10.1186/s12888-017-1287-1) contains supplementary material, which is available to authorized users.

## Background

People with severe mental illness suffer higher rates of multiple, often more complex, physical comorbidity and premature death than the general population [1]. As a result, life expectancy for this vulnerable population is reduced by 15–20 years [2]. It is well established that side effects of some antipsychotic medications contribute substantially to this inequality alongside other factors such as, higher rates of smoking, substance abuse, poor nutrition and sedentary lifestyles [3].

Last year, the independent mental health Taskforce set out a national strategic vision to deliver a Five Year Forward View to improving mental health outcomes across the health and care system in England by 2020/21 [4]. The strategy is the result of engagement with over twenty thousand people with lived experience of mental health conditions, families, carers and professionals, as well as a review of clinical and economic evidence [4]. The Implementation Plan aims to provide improved continuity and coordination of care, and enhanced relational support between health and social care or between primary and secondary care service providers by bringing together physical and mental health care to benefit people with severe mental illness [4]. In response to the Taskforce, promises have been made to deliver more integrated physical and mental health care, to provide NHS care that is cost efficient, of a higher quality, and more personalised to service users of mental health services [5, 6]. Yet, despite efforts to improve mental health care, a number of factors continue to make it difficult to provide an integrated response to service users physical and mental health needs, including a lack of institutional time and space to be able to offer more personalised care; cultural barriers at team levels to enable improved assessment, treatment and communication between professionals within mental health services; and separate systems for physical and mental health care delivery [5]. Mental health professionals’ commonly report having insufficient skills, competencies and resources to perform physical health checks in addition to mental health checks [3]. As a result, many physical health checks, such as vital cholesterol and blood glucose checks to prevent the onset of long-term physical health conditions, are not completed by mental health professionals, with many service users being referred on to physical health specialists in primary care [3, 7].

One approach mandated to enable decisions to become tailored to the individual service user and carer to significantly improve health outcomes is individual care planning [8, 9]. In the UK, the recovery-focused Care Planning Approach (CPA) is mandated nationally to assess service users mental and physical health needs [10–12]. In line with the CPA, service users and carers should have access to a care coordinator, a written care plan and an annual review to check their progress. Service users should work collaboratively with their care planning team to discuss and document their plan of care [9, 13, 14]. Furthermore, service users are expected to take joint responsibility for achieving their goals reflecting the core principles of shared decision-making [9, 14, 15]. Typically, a care plan, or a CPA review letter, should be the outcome of these discussions, and should accurately document the interplay between an individual’s mental and physical health symptoms, all primary and secondary mental health and physical health diagnoses and treatments, to ensure essential and current information necessary for high quality care of a service user of secondary mental health services is recorded and communicated to primary care [16].

Qualitative research has highlighted how service user and carer led involvement in mental health care planning persists as an under-performing area. Concepts of user involvement have been shown to be operationalised differently by service users and mental health professionals [17]. In practice, poor information exchange between mental health care professionals, insufficient resources to empower service users and carers, and conflicts between mental health care professionals, frequently lead to decisions being taken without service users and carers involvement [18].

Whilst there is some empirical evidence that coordination of care and maintaining a relationship is considered to be central to user-led involvement in mental health care planning [19], it remains unclear how physical health is prioritised and operationalised within this process. This knowledge gap is exacerbated by the lack of clarity over where the responsibility for providing physical health care to service users lies. Debate continues as to whether this rests principally with mental health professionals working within mental health services, primary care, or both [2, 5, 20–22]. Both service users and mental health professionals have reported difficulties with accessing appropriate physical health services, with the shared concern that physical and mental health needs are still being addressed by mental health services in a disconnected way [5].

Parity of esteem is a health policy concept that advocates equal value for mental and physical health services. This includes the priority and attention given to both in research and practice. The aim of this paper is to explore stakeholders’ experiences and preferences of service user and carer led involvement in physical health care discussions within mental health care planning. Recent studies in generic mental health care planning have revealed how the conceptual framework underpinning user-involved care planning in mental health services may differ from the patient centred care framework adopted in physical health services [23]. This work emphasises the need to recognise the cultural and contemporary contexts in which service users engage in care planning within secondary mental health services. These contexts have traditionally led to more paternalistic approaches to care planning, which are a real barrier to adopting and practicing service user led involvement [18, 23].

Understanding how service users and carers feel involved in planning their physical health care remains a key step towards improving levels of preference-guided involvement in mental health care planning [23]. The current study addresses the aforementioned gap, through understanding the experiences and preferences of service users, carers and professionals, in relation to their preferred levels of involvement in physical health care discussions within mental health care planning.

The study used an explorative approach to inform the development of a conceptual framework of service user and carer involvement in physical health discussions within care planning. This framework offers a useful foundation to begin to understand how user-led involvement in physical health care discussions is achieved in practice from multiple stakeholder perspectives [23], with a view to expediting a move towards parity of esteem between mental and physical health services, and improving the physical health of people with serious mental illness.

## Method

### Study design

The study utilised a qualitative approach, incorporating focus groups and semi-structured interviews, to explore how the concept of user involvement in physical health discussions within mental health care planning is experienced from the service user, carer and mental health professional perspective, to develop a conceptual framework of effective user-led involvement. This approach was taken because we still have a limited understanding of how this concept is understood in this area of service provision.

From herein, our study methods are reported in line with the consolidated criteria for reporting qualitative research (COREQ) guidelines [24].

### Participants

Service user, carer and mental health professional participants were recruited from Manchester Mental Health and Social Care Trust (MMHSCT) in the North West of England, UK. At the time of our study, the MMHSCT was the main provider of specialist mental health care, social care, and health and wellbeing services to the residents of Manchester, supporting over 14,000 service users per annum, with a workforce of over 1500 and an annual income of over £104 m [25].

Stakeholders’ were recruited from MMHSCT to compare experiences of service user and carer led involvement in order to highlight any differences in perspectives and to inform the conceptual understanding of effective service user and carer led involvement. Thus, mental health professionals were recruited, as well as service users and carers, to discover how they experienced service user and carer led involvement in routine practice, and to establish preferences of service user and led involvement from all stakeholders involved in the process of physical health care discussions within mental health care planning.

Participant recruitment and data collection occurred between June and September 2015. Participants were recruited using a range of established recruitment methods [26, 27], to attract service users, carers and mental health professionals to take part in the study, via: the Trust intranet and communications, posters displayed on Trust and University premises and service user and carer forums.

Inclusion criteria comprised service users who were aged eighteen or over with current or recent involvement with community mental health services. Carers were eligible for inclusion if they were caring or supporting a person with a serious mental illness who had experience of secondary mental health services. Mental health professionals had to be working in community mental health services within the specified Trust.

### Data collection

Data collection took place either on the University of Manchester or Trust premises. Focus groups were used to encourage open-ended group interaction and user-led dialogue to elicit experiences and preferences of involvement in physical health care discussions within mental health care planning.

Potential participants contacted the study coordinator (NS) to discuss study eligibility, and interview availability. Following this, participants were invited to attend a focus group, or to take part in a telephone interview at a convenient date. This choice was mostly determined by participant preference. Participants took part in either a focus group or an interview, not both. Eligible participants were sent (via email, or post) a study information sheet. Before the start of each focus group or telephone interview, participants were asked if they had any further questions prior to giving written consent. Participants who chose to be interviewed on the telephone were subsequently sent a study written consent form and a self-addressed envelope. Interviews and focus groups were arranged on receipt of written consent. All interviews and focus groups were digitally recorded with written consent.

The research team consisted of health services researchers, a service user researcher, and a clinical member of the Trust concerned. Thus, our team was multidisciplinary with significant, methodological, service user lived experience, clinical, psychometric and academic expertise. We followed a topic guide which provided a flexible and established framework [23], to understand experiences and preferences of service user and carer preferred levels of involvement in physical health discussions (see Additional file 1). Questions specific to physical health were incorporated in a topic guide; questions specific to service user and carer involvement were informed by a previous generic mental health care planning topic guide which had been ‘user tested’ by a service user and carer advisory group (SUCAG [23]). We defined ‘physical health’ as an essential part of a service user’s overall health, and included everything from their physical fitness and function, to their overall physical wellbeing. Topics explored covered current perspectives of physical health care discussions, including: a) the importance of physical health in relation to mental health; b) the process; c) the outcome; and, d) perceptions of service user and carer involvement (see Additional file 1 for detail).

The majority of the focus groups and telephone interviews were facilitated by the study co-ordinator (NS); the minority were facilitated by a CLAHRC for Greater Manchester PhD student (IA). All focus groups were co-facilitated by one of two members of the research team (HB, RP). All facilitators and co-facilitators self-identified as experienced health services researchers without professional or personal experience of secondary care mental health services. Each focus group lasted on average 66 min (range: 50–89 min); telephone interviews were shorter averaging at 34 min (range: 29–36 min). Service users and carers were compensated for their time with a payment of a high street gift voucher (£25) and travel expenses were reimbursed. Once each focus group or telephone interview was completed, the topic guide was adapted in line with the data collected and new avenues of interest were explored in the next interview or focus group.

A total of forty seven participants expressed an interest in participating in the study, and thirty four participants consented and took part in either a telephone interview or a focus group. From our three groups of participants, focus groups were undertaken with: twelve service users; nine carers; two service users with a dual service user and carer perspective; and seven mental health professionals working in different roles within mental health services. Of note, two mental health professionals were also service users, but chose to participate in a professional focus group. Telephone interviews were undertaken with three mental health professionals, and one service user and carer (bringing a dual perspective).

Table 1 presents the sample characteristics of the participants; the majority of our participants were female service users.

**Table 1:** Sample characteristics relating to participants

Interview type (Number)	Participant type (Number)	Total number of participants	Gender	
			Male	Female
Focus group (6)	SU (12), SU/C (2), C (9), MHP (5), MHP/SU (2)	30	8	22
Telephone (4)	SU/C (1), MHP (3)	4	2	2

Key: *SU* Service user, *SU/C* Service user and carer dual perspective, *C* Carer, *MHP* Mental health professional, *MHP/SU* Mental health professional and service dual perspective

### Data analysis

Interview data was transcribed and anonymised upon receipt. The analysis process was led by the study coordinator (NS); all members of the research team contributed to the analysis by reading a selection of transcripts, coding text segments and offering suggestions to the developing framework. All members of the research team verified the final set of themes.

Data was subjected to a thematic analysis, whereby emerging themes were generated, coded, re-coded and categorised accordingly [28]. As coding progressed, an excel spreadsheet listing the codes, and a document containing codes from each transcript with excerpts of data relevant to each theme, were developed to manage the developing thematic framework. All codes were checked by revisiting the transcripts to ensure that the emerging themes remained grounded in the service user and carer perspective, and audio files were checked to assure the quality of transcription.

Data from the service user and carer perspective were coded separately from the professional data, and subsequently compared to allow for comparison of experiences. During the comparison of data, the framework was reshaped to enable new themes to be introduced. The research team agreed as a whole when data saturation had occurred (that is, the point at which no new themes emerged from the data), and no further data collection was necessary.

The analysis identified key prerequisites of effective service user and carer involvement in physical health discussions within care planning, and a conceptual framework that represented the entire data set was developed. Data are presented to compare and contrast service user, carer, and mental health professional experiences of service user and carer led involvement in physical health discussions within mental health care planning.

## Results

**Fig. 1:**
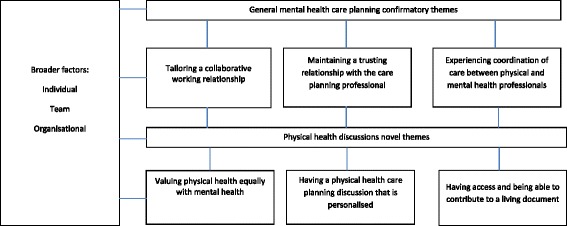
Conceptual framework: six prerequisites for effective service user and carer led involvement in physical health care planning and broader factors that impact on the implementation within mental health services

Six prerequisites for effective service user and carer involvement in physical health discussions within care planning emerged from the data; three of which confirmed general care planning requirements (tailoring a collaborative working relationship, maintaining a trusting relationship with the care planning professional and having access to and being able to contribute to a living document) and three which were novel themes, or those that had specific relevance to physical health discussions (valuing physical health equally with mental health, experiencing coordination of care between physical-mental health professionals, having a physical health discussion that is personalised). The conceptual framework detailing these is presented in Fig. 1.

Each prerequisite is presented in relation to the current perspectives, processes and outcomes for effective involvement, and the broader healthcare system factors that impact on the implementation of effective user and carer led involvement. Illustrative data are given and identified by a code number rather than a name or pseudonym, which is provided along with contextual information on the interview type and participant group.

### Themes confirming general care planning requirements

#### Tailoring a collaborative working relationship

Tailoring a collaborative working relationship between professionals and those people with lived experience was described within accounts as a prerequisite for effective service user led involvement in physical health care discussions within mental health care planning. Through a collaborative working relationship, there was the potential for service users and carers to feel empowered, and more confident to become involved in tailoring their physical health elements of the care plan. There was also reference to how collaboration between mental and physical health professionals, and the people who use the services, might help to tailor the wider health and social care provided to the needs of patients with serious mental illness, emphasising people’s preferences to move towards integrated physical and mental health care.

*“I think that one of the best things that users and carers can bring is experts by experience, and they can really help to shape the whole, kind of, how our health and social care system works, to make it more integrated”* [5641, telephone, service user and carer].

Likewise, professionals described how the successful facilitation of a collaborative working relationship was a two-way process. This involved service users taking ownership of care planning, and professionals establishing some context about service users’ level of motivation and commitment to the process, to tailor the collaborative working partnership. Collaborative mental health care planning had potential benefits for both parties if realised effectively.

*“It’s a two-way process… it’s something that they’re involved with and taking part in and contributing to, rather than something that’s being done to them… they [professionals] might learn the reasons why people do things in certain ways and be able to manipulate something around that. And having that full picture of that patient”* [6225, telephone, mental health professional].

Across stakeholder accounts, the potential for collaborative working was often missed due to broader factors within the healthcare system, such as; time, resources, workload constraints, service users characteristics, and carer-patient confidentiality.

*“I think a lot of patients are quite passive in themselves about some important health priorities, and if they are passive about those and there are some other difficulties such as time constraints, resources in the health and social care system, that passivity combined with lack of resources does conspire to neglect kind of assertive physical health care plans”* [6372, telephone, mental health professional].

Carers highlighted the value of their role within collaborative care planning discussions because of the in-depth knowledge they had about the service user they care for. This value was considered particularly relevant in situations where service users were less involved in care planning. Carers’ perspectives resonated with both the service users’ and professionals’ preferences for tailoring collaborative working relationships to the individuals involved, thereby optimising the likely relevance and implementation of care recommendations.

*“…there’s a huge assumption that the cared for will be able to take up that offer of physical health, and I think that’s a huge assumption for that person who’s not well to be able to suddenly skip to the gym or do whatever. And I think without the role of the carer it’s just not going to happen… I’m sure there’s many people out there that might be given social prescribing, and it does nothing for them because they’re not able to actually make that step to actually do it. I’d make sure the cared for actually goes to the gym or whatever, which is actually quite a challenging task in itself sometimes*” [5277, FG3, carer].

#### Maintaining a trusting relationship with the care planning professional

Service users felt that maintaining a reciprocal trusting relationship with their mental health professional was a prerequisite to feeling able to attend, and contribute to care planning discussions about physical health. Service users and carers described the need to feel that their mental health professional acknowledged what they were saying and trusted them, so that they would feel able to openly discuss their physical health needs. Having an established two-way trusting relationship was also reflected within professional accounts.

*“I think, for a start, it’s the sense of knowing that nothing is physically wrong with me, or when it is, knowing that I’m getting the right treatment or I’m not being misunderstood by a GP or other form of professional, that it’s just the mental health that’s making you think like that”* [5641, telephone, service user and carer].

*“It’s having somebody visible that can go and access and to take that information on board and having somebody there for when they need that information. But it’s building up that baseline first that people know that there’s people there that they can access. It’s knowing what resources they can tap into”* [6225, telephone interview, mental health professional].

Without this trusting relationship, service users and carers reported finding it difficult to raise or discuss sensitive issues within consultations. For example, sexual dysfunction and weight gain associated with antipsychotic medication were common physical health concerns that service users wanted to raise with professionals. However, they did not feel they could do this with a professional they did not trust.

*“I think trust is a big issue... You know, you get a number of therapists that make people feel like freaks, you know, and there’s not going to be any positive remark... especially if you talk about things like smoking that people enjoy, or anything that’s hard to change, you’re not going to do that with someone you don’t trust” [*5276, FG2, service user and carer].

Despite these potential benefits, professionals’ described how time and skill-mix constraints within the mental health workforce were barriers to developing these relationships. There was also an acknowledgment that a trusting relationship would need to be built over time requiring a level of continuity of care that was not possible within current mental health services.

#### Having access to and being able to contribute to a living document

Having access to a living document with tailored content and regular follow-up was the preferred outcome to having a physical health discussion that was personalised. Ideally, a care plan would be a holistic document including tailored content related to both mental and physical health which service users could access and edit, helping to maintain its currency, moving it from a reactive document to a proactive document.

*“I don’t even want to call it physical or mental, I’d rather just call it a wellbeing health plan or a wellbeing plan, because both parties shouldn’t ignore the other part of your life, you know, they are both equally important. If I go to see a physical health practitioner, I would like that physical health practitioner to ask me questions around how am I feeling, what is my mental state like, what impact is this physical health condition having on me? And I would like the same for a nursing staff to do in a mental health setting, to ask me about my physical health. So I think that for me there should be something that people should see you as a whole person”* [5641, telephone, service user and carer].

There was a perception amongst service users and carers that current care plans were written from the perspective of professionals to serve professional and organisational requirements.

*“I don’t have one [care plan]… if one was written it would be written for them [mental health professionals’], by them without anything to do with me. So I mean I wouldn’t see it anyway. So I mean the things I want, or if I had an imaginary care plan, is a discussion about what affects me, you know, like a sort of level of… what’s having the biggest impact on me and maybe where I can get support with these things, that would be the kind of thing I’d want”* [5276, FG2, carer].

Many service user and carers described not having received a care plan, nor did they recall direct involvement in physical health discussions within the care planning process. If a care plan existed, service users and carers were not necessarily involved in developing the content, nor were they able to access or edit it. Of note, all participants’ emphasised that even for those physical health discussions that did occur, these were not always reflected on and updated in the care plan. As a result, the document remained static and unhelpful to any subsequent physical health discussions, or treatment.

*“I haven’t seen a care plan for eighteen month, maybe two years. It must be approaching two years that my social worker said ‘oh, I’ve done your care plan, will you have a look at it for me’. These things need updating every three to six month at the very least”* [5275, FG1, service user and carer].

### Novel themes with specific relevance to physical health discussions

#### Valuing physical health equally with mental health

A core prerequisite to establishing effective service user and carer led involvement in physical health discussions, which appeared across participant group accounts, was the importance of recognising the parity of physical and mental health in the context of a holistic approach to physical and mental health care planning within mental health services. A holistic approach was perceived by all participants’ to be consideration of the whole person’s physical, emotional, social, and spiritual health.

*“So it is important that it is connected, the mind and body, because what you think and that, it affects what you eat and everything. But it’s important to treat them both as equal and the same” *[5277, FG3, carer].

However, participants generally reported a lack of holistic approaches to mental health care planning for a number of reasons. Professionals described how mental health services are siloed from physical health services with current health service structure and provision. There was also a tendency for mental health professionals to treat physical health as a second priority to mental health. Of particular note, prioritisation of mental health over physical health was highlighted across participants’ accounts describing particular experiences whereby mental health professionals’ minimised physical symptoms, which evoked feelings that physical health concerns were not valued equally with mental health.

*“I think fundamentally commissioning is where we’re going wrong with physical health, getting it together with mental health and we look at parity of esteem and I think mental health services are working towards parity of esteem, we are starting to look at physical health as something we do, we’ve got to get it right, it’s really important, it doesn’t work the other way, the acute trust do not, when you’re having your appendix out, consider your schizophrenia or your medicines”* [5427, FG5, mental health professional].

*“I’ve had a personal experience where you explain a condition to a doctor and he just doesn’t actually realise that what you’re saying is physically happening, he just thinks it’s just part of your mental illness and they kind of fob it off”* [5641, telephone, service user and carer].

Service users and carers frequently complained about professionals taking limited responsibility for their physical health concerns, and subsequently referring them to physical health specialists outside of mental health services. Many mental health professionals acknowledged this referring of service users onto physical health services, most commonly the GP. They described not having the specialist physical health knowledge, or access to onsite physical health services to treat physical health effectively within mental health services.

*“I think staff will often say, well, you need to go to the GP. And so a lot of them redirect them to the GP really, if there’s a physical health problem. Because they don’t have the knowledge, primary care’s the best one, the GP has the knowledge”* [6390, Telephone, mental health professional].

Despite these descriptions of separation of mental and physical health within health service structure and provision, all participants’ described an overwhelming difficulty in separating the experience of physical and mental health symptoms. This was particularly common when service users and carers experienced side effects, such as weight gain, from antipsychotic treatment.

*“I think in relation to having a mental health diagnosis, for me physical needs to be part of the equation, because most people get put on medication where the side effects aren’t explained fully, so you’re experiencing different physical things that you’ve probably never encountered before. So the physical aspect needs to be included to include them side effects”* [5275, FG1, service user and carer].

#### Experiencing coordination of care between physical-mental health professionals

A prerequisite of effective involvement was experiencing coordination of care between mental and physical health professionals. Integrated care was described by service users, carers and mental health professionals as an approach to health and social care that needed to happen for effective user and carer involvement in physical health care discussions within the care planning process to be implemented in mental health services.

*“What I would like more of, is that there’s more coordination in my mum’s care plan around physical practitioners, healthcare practitioners working along with mental health practitioners”* [5641, telephone, carer].

In order to realise the level of coordination of care required, professionals’ described needing more physical health resources within mental health services in order to deliver coordination of care, such as locating hospitals next to community mental health hospitals, and building physical health clinics within mental health services.

*“I think we need the resources ourselves really. The Community Mental Health Trust needs to provide that. When people come to an outpatient appointment… the consultant sends a letter to the GP and says, can you do the bloods and the ECG. Now that doesn’t always happen. And we don’t know if it happens, nobody… we just have to ask the patient. So if you had somewhere there like they do at [name]… I know they’ve got a physical health clinic there and people go to an outpatient appointment, maybe they could just pop in and have their bloods done, and they can have an ECG or they can have an appointment to come back and have a physical health check. I think we need something like that”* [6390, telephone, mental health professional].

#### Having a physical health care discussion within care planning that is personalised

Having a physical health care discussion within the care planning process that is personalised to the service user was a prerequisite to feeling involved in the process and outcome of care planning as described by service users and carers. Service users and carers, however, reported that no specific physical health care meetings took place within mental health services. Participants felt that when physical health did feature in general mental health care planning discussions, this represented a ‘tick box’ exercise focussed on specific health conditions or organisational requirements, rather than a meaningful discussion personalised to the individual’s holistic physical health needs.

*“They are discussed. There’s no doubt about that, I wouldn’t say that they don’t get discussed because, you know, part of my care plan when I used to get a lot of services was input around my mental wellbeing. They used to cover, you know, all the physical aspects of it, but it was a tick box exercise. It was very much a tick box exercise: ‘okay, let’s da-da-da-da’, it would only… But, actually, when you have something, no thorough detail no thorough interest is taken by mental health practitioners”* [5641, telephone, service user and carer].

Professionals felt that planning care for physical health needs within the care planning process was a longitudinal process and that in practice mental health services had yet to achieve equilibrium between mental and physical care planning needs due to individual and broader healthcare factors. Professionals described how physical health concerns were often discussed in relation to the structured questions in the Rethink Physical Health Check tool in line with the CPA [29]. This ensured that physical health discussion remained focused. However, sole reliance on this tool resulted in no room for expansion of the physical health discussion to meet the service users’ individual care planning needs and might lead to service users feeling that physical health care discussions were not meaningful.

*“I think the care planning, the patients themselves need to understand why it’s done, it’s not just a tick-box exercise [using the rethink tool], that it has actually got outcomes, and it’s for their health. But it’s them perceiving that… care planning takes a bit longer. When you involve the patients and you take them on board, there are things that will be raised up and how you actually signpost that person to the more appropriate place, will they take up more time in the extra help that they potentially might need to support them through this”* [6225, telephone, mental health professional].

## Discussion

People with severe mental illness suffer more complex physical comorbidity than the general population, requiring a more tailored approach to care planning from mental health services. The present qualitative study sought to expand on policy mandates for integrated physical and mental health care in the NHS [30], by defining best practice for physical health planning for service users across individual, practice and organisational levels. This study is to our knowledge, the first empirical research study to present a conceptual framework of high quality physical health discussions for mental health service users, providing a much-needed platform for service improvement and future research and development activities. Our data suggests that a more tailored approach to physical health care discussions within mental health care planning, which looks at the whole person in context with their unique needs and personal characteristics, is necessary for effective service user and carer led involvement to be achieved in practice.

The novel contribution to our study lies in the development of a unique conceptual framework encompassing the prerequisites necessary to achieve effective service user and carer involvement in physical health care discussions; this work provides a foundation to understand how the concept might be translated into practice. Our evidence-based framework highlights the relationships between the different prerequisites, and how real service user and carer involvement might be achievable through the measurement of those prerequisites (see Fig. 1). We have shown that service user and carer involvement in physical health discussions is influenced by different factors, and is expedited by and judged according to six different domains of activity: valuing parity of esteem; tailoring a collaborative working relationship; maintaining a trusting relationship; experiencing coordination of care between physical and mental health professionals; having a physical health discussion that is personalised; and being able to contribute to the outcome of those physical health discussions in the short and longer term through accessing, and editing a living document that encompasses a current plan of physical and mental health wellbeing. In further work, we are now in a position to operationalise our conceptual framework, by developing a new patient reported outcome measure (commonly known as a PROM) of service user and carer led involvement in physical health care domains of mental health care plans. Psychometric PROMs are reliable and valid measures used in translational research in the NHS to quantify domains of interest, to enable meaningful measurement of health care quality and patient experience outcomes from the service user perspective [23].

A strength of our approach is that we developed our conceptual framework through consultation with service users and carers to identify their perspectives, and combined this data with insight from the mental health professional perspective with experience of operationalising service user and carer led involvement in physical health care discussions within mental health care planning. Our data reveals a need to improve the relational aspects underpinning physical health care discussions, in order for meaningful service user and carer involvement to occur. Importantly, we have identified a conceptual model of ‘best practice’ care planning that challenges current practice [11], having found no evidence within our study that service users and carers had been meaningfully involved in physical health care discussions; some participants reported receiving no care plan at all.

We have shown that efforts to improve the physical health care of people with serious mental illness should be developed in consultation with the stakeholders to empower service users, carers and mental health professionals, to help remove barriers to delivering and accessing more integrated care within mental health services. Of note, we found no evidence of examples where stakeholders have had input into the development of integrated care systems. Therefore, our study has highlighted the need to develop more bespoke and meaningful interventions for people with serious mental illness, to be able to treat physical health care with equal importance as mental health care, coordinate care between physical-mental health professionals, and have physical health discussions that are personalised to the user. Further, our three novel themes illustrate how service users require their physical health, mental health and social care to be coordinated around them, and for this to happen, this requires a partnership with service users and carers over the long term, rather than providing unconnected care. Thus, our framework emphasises the importance of providing an integrated approach to the identification, assessment and support of service users physical health needs across health and social care [5, 30].

Our data has some conceptual similarities to evidence-based frameworks developed for user-carer led involvement in generic mental health care planning [17, 18, 26, 27]. Within mental health care planning, service users, welcome the partnership element of collaborative working, and valued relational aspects of care planning and request a personalised approach to care [17, 18, 27]. Our data suggests that service user dissatisfaction with physical health planning may be lessened by personalising physical health discussions and resources [26, 27]. Likewise, feelings of isolation and vulnerability would lessen as service users and carers become more involved in the care planning process at a preferred level of involvement [26, 27, 31].

Operationalising such a process within mental health services demands action and cultural change at team and organisational levels. Staff time was a clear barrier that impacted on the quality, depth and meaningfulness of those personalised service user and carer led care planning discussions [17, 27]. Moreover, the potential for service user and carer led involvement was often missed due to other broader factors within the healthcare system, such as; resources, workload constraints, and organisational barriers within the mental health setting [17, 18, 27]. A crucial organisational barrier in our findings was the dilemma and lack of clarity around service responsibility - mental health or physical health services - for provision of physical health care. Interestingly, this reflects previous International qualitative research whereby mental health and primary care health care systems have been found to operate in silo, resulting in no clear preference as to which physical-mental professional should provide the physical care [3]. However, our unique framework with specific relevance to physical-mental health domains may help to resolve this recurring dilemma for mental health professionals through ensuring transparency of service responsibility via coordinating care with physical professionals, and documenting those meaningful physical health care discussions and outcomes on a living care plan that either professionals may consult to clarify provision of physical health care, and to give context on their physical health needs.

We have identified a need for specific workforce physical health training to ensure that physical health care delivery was fully and effectively integrated within mental health services. Professionals in our sample described a lack of confidence and skill in dealing with service users physical health, leading to a need to ‘refer on’ to other more skilled physical health professionals outside of mental health services [3, 7, 18]. As a result, many service users and carers described being ‘fobbed off’ by their mental health professional, or being ‘passed about’ to a physical health specialist without knowledge of their mental health difficulties, or recovery journey, which in turn impacted on service users and carers motivation levels to be involved in any subsequent physical health discussions. The aforementioned barriers to service user and carer led involvement in physical health discussions in mental health care planning resonate with the recent mandate to bring together physical and mental health care as a third dimension to health and social care in the NHS [5]. Initiatives to overcome these barriers and implement integrated physical and mental health care recommend professional bodies to redesign curriculums so that all health professionals have a common foundation in mental health, as well as physical health [5]. This may overcome the dilemma of prioritisation of mental health over physical health which service users/ carers and mental health professionals described, as well as mental health services not taking shared responsibility for physical health concerns. Other initiatives mandated involve providing ongoing training for mental health professionals to closely monitor physical symptoms, as well as training to improve communication to GPs, to ensure integrated models of care are in place to sustain good practice [16].

Our data substantially strengthens the mental health Taskforce 5 year objective to increase investment in mental health funding, to achieve a system-wide transformation for mental health services to be able to achieve parity of esteem [5, 30]. Ultimately, our data highlights how mental health services still have a long way to go to achieve the cultural change to deliver the prerequisites necessary to bring about true service user and care led involvement in care planning discussions [5]. However, our conceptual framework centred on valuing parity of physical and mental health, coordination, personalising physical health discussions and documents to reflect the service users and carers holistic and individual needs, may assist the delivery and embed service user and carer individual care planning in practice, against achieving the Taskforce 5 year vision of offering more preventive, and holistic mental health care [4].

Of note, the integration of Greater Manchester West Mental Health NHS Foundation Trust and MMHSCT to form Greater Manchester Mental Health NHS Foundation Trust (GMMH) took place two years on following the completion of our study on 1^st^ January 2017, to improve prevention, access, integrated approaches towards physical and mental health and the sustainability of Manchester’s mental health services [32]. This represents the first phase of the wider transformation of mental health services across Greater Manchester, which will see the integration of social care, primary care and mental health provision together at the community level, and mental health services collaborating around specialist provision of care [32, 33]. The new pooling of budgets to enable joint decision making as an integrated whole is thought to allow new models of care to be developed and supported to improve the health and wellbeing of service users and carers [33]. Our platform for best practice for effective service user and carer involvement in physical health care planning complements the new strategic vision of the GMMH Trust for the future delivery of mental health services across Greater Manchester, with particular investment in stakeholder improvement schemes across the Trust, designed to improve physical and mental health service user experience, and integrated health and social care.

### Limitations of the current study

There are limitations to the findings that we have presented. Given the nature of our approach to recruitment, a few of the participants had participated in a previous user involvement focus group as part of the wider involvement in care planning programme; thus, previous experience may have influenced accounts given [23]. The range of characteristics of participants in the study was limited by the opt-in methods; we sampled more female participants, and more service users than carers. We collected limited demographic characteristics (gender, participant type and role), but we intend to collect further demographic data from a larger sample of service users and carers when we develop and preliminary test our new PROM in future work. The potential for purposive sampling in responses to developing themes was minimal given the nature of the opt-in method to recruitment. The ethical requirement to obtain written consent from all participants impacted on the number of mental health professionals recruited to be interviewed over the telephone; many participants failed to return written postal consent. The diagnosis of serious mental illness was not verified by a mental health service provider that they were using, or the GP, and represent people’s perceptions. It is possible that these sampling issues may have led to a restriction in the range of interpretation of findings if certain types of participants do not respond. For instance, those patients who may not have a good relationship with their care planning team may have decided to not take part. The findings may not be generalizable to all types of serious mental illness and physical health conditions; and type of condition is a variable to explore in our further research. We did not collect baseline data on professional role. Professionals were self-identified as being involved in physical health discussions with mental health service users and carers. However, professional participants were primarily recruited to explore how involvement was operationalised in practice, to complement and contrast, service user and carer experiences of involvement.

## Conclusions

We identified six prerequisites for effective user-led involvement in physical health care discussions within mental health care planning and organised them into a conceptual framework of confirmatory and novel themes. Our data revealed that user-led involvement in physical health care discussions is an interpersonal process that if facilitated correctly, should empower service users and professionals to work together effectively. Improvements in physical health discussions within the care planning process, and the delivery of integrated care, demand action at individual, team and organisational levels. A conceptual framework is presented which provides an evidence-based foundation for service level improvement. In our future work, service user and carer centred items will be developed for a new PROM to quantify and move towards implementing service user and carer led involvement in practice.

## Additional file

Additional file 1: Participant data collection topic guide. (DOCX 186 kb)

## Abbreviations

CLAHRC: Collaboration for Leadership in Applied Health Research and Care for Greater Manchester
COREQ: Consolidated criteria for reporting qualitative research guidelines
CPA: Care Planning Approach
ECG: Electrocardiogram
GMMH: Greater Manchester Mental Health NHS Foundation Trust
GP: General practitioner
MMHSCT: Manchester Mental Health and Social Care Trust
PROM: Patient reported outcome measure
SUCAG: Service user and carer advisory group
