# Supplementary material for: Understanding experiences of and preferences for service user and carer involvement in physical health care discussions within mental health care planning
Source: BMC Psychiatry. 2017 Apr 13;17:138. doi: 10.1186/s12888-017-1287-1 (PMC5390472; doi:10.1186/s12888-017-1287-1)
Supplement: Additional file 1: — Participant data collection topic guide. (DOCX 186 kb) [file 12888_2017_1287_MOESM1_ESM.docx]

**Additional File 1: Focus Group & Telephone Interview Schedule:**

**Users/Carers/Professionals**

**Structure**

1. Welcome & Study background
2. Purpose of group and ethical considerations/provision
3. Timetable & Tasks
4. Ground rules (including confidentiality, respect for other people’s opinions, free to answer question or not etc.)
5. Getting to know each other

**Current Perspectives**

- What does ‘physical health’ mean to you?
  - What aspects of physical health are most important to you?
  - What would you most like help with?
- What does ‘care-planning’ mean to you?
  - Is it an easy process to understand?
- In your experience, what are the attitudes of staff in mental health and social care services to care planning for physical health issues?
  - What helps?
  - What hinders understanding?
  - Is it easy to express lack of understanding?
- Do you think that a care plan influences the nature of physical health care that users receive?

**Current Processes:**

- In your experience of care planning for physical health issues:
  - Do ‘staff listen to the concerns of patients?’
  - Do staff respect the knowledge & expertise of users/carers?
  - Is there sufficient ‘patient involvement in making decisions about care’?
- Thinking about the role of the care coordinator, what assistance have they given you to help your physical health [revise for professionals, what assistance have they given service users/carers to help them with their physical health?]
  - What else could they/should they have done?
- What is the most important thing users and carers bring to physical health care planning?
- What information do users get about physical health services?
- How frequently do care planning meetings for physical health take place?
  - Who normally attends
  - Where do they take place?
  - How suitable is attendance/location for you?
- What happens at these meetings (structure and content)?
- What is the communication style of the meeting?
  - Could this be improved in any way?
- What aspects of your physical health do you think needs to be recorded on a care plan? Why?
- Do you know what happens to a care plan once it has been completed?
  - Do service users know how to access the care plan? Where is it stored?
  - Who uses it?

**Outcomes:**

- Do care plans for physical health have a useful impact?
  - For users/carers?
  - For mental health/social care organisations?
- Can you share any examples of good outcomes as result of care planning?
  - Any bad care planning outcomes?
- What are the essential ingredients for good physical health care planning?
- What information on care planning would be useful for users and carers?
- How good are different mental health and social care professionals at care planning for physical health?
  - Any differing experiences with different professional groups?

**User Involvement:**

- Do you users and cares feel involved in care planning processes?
  - Do you/they want to be involved?
  - What do you think would be a suitable level of involvement?
- What might be the benefits of involving users/carers in care planning for physical health?
  - What might staff learn from users/carers?
- Could there be any disadvantages to increasing user/carer involvement?
- What support would users and carers need to become more fully involved with care planning for physical health?
  - Knowledge?
  - Resources?
